# InGaP *χ*^(2)^ integrated photonics platform for broadband, ultra-efficient nonlinear conversion and entangled photon generation

**DOI:** 10.1038/s41377-024-01653-5

**Published:** 2024-10-15

**Authors:** Joshua Akin, Yunlei Zhao, Yuvraj Misra, A. K. M. Naziul Haque, Kejie Fang

**Affiliations:** 1 Holonyak Micro and Nanotechnology Laboratory and Department of Electrical and Computer Engineering, University of Illinois at Urbana-Champaign, Urbana, IL 61801 USA; 2 Illinois Quantum Information Science and Technology Center, University of Illinois at Urbana-Champaign, Urbana, IL 61801 USA

**Keywords:** Nonlinear optics, Quantum optics

## Abstract

Nonlinear optics plays an important role in many areas of science and technology. The advance of nonlinear optics is empowered by the discovery and utilization of materials with growing optical nonlinearity. Here we demonstrate an indium gallium phosphide (InGaP) integrated photonics platform for broadband, ultra-efficient second-order nonlinear optics. The InGaP nanophotonic waveguide enables second-harmonic generation with a normalized efficiency of 128, 000%/W/cm^2^ at 1.55 *μ*m pump wavelength, nearly two orders of magnitude higher than the state of the art in the telecommunication C band. Further, we realize an ultra-bright, broadband time-energy entangled photon source with a pair generation rate of 97 GHz/mW and a bandwidth of 115 nm centered at the telecommunication C band. The InGaP entangled photon source shows high coincidence-to-accidental counts ratio CAR > 10^4^ and two-photon interference visibility > 98%. The InGaP second-order nonlinear photonics platform will have wide-ranging implications for non-classical light generation, optical signal processing, and quantum networking.

## Abstract (web summary)

We demonstrated a novel InGaP integrated photonics platform for ultra-efficient, broadband second-order nonlinear optics, including time-energy entangled photon generation, transcending the current state of the art.

## Graphical abstract

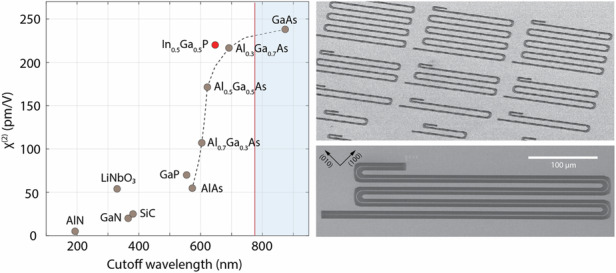

## Introduction

The development of nonlinear optics is empowered by the invention of nonlinear materials, from bulk nonlinear crystals and silica fibers to more recent wafer-scale thin-film materials. Over the past decades, the application of materials with increasing nonlinearities, combined with the advance of light-confining nanophotonic structures, has resulted in a remarkable enhancement in nonlinear optical efficiencies. For example, the second-harmonic generation has advanced from the initial demonstration using a quartz crystal with a 10^−9^%/W efficiency^1^ to the record of 10^5^ − 10^6^%/W achieved in thin-film nanophotonic resonators nowadays^2,3^.

**Fig. 1:**
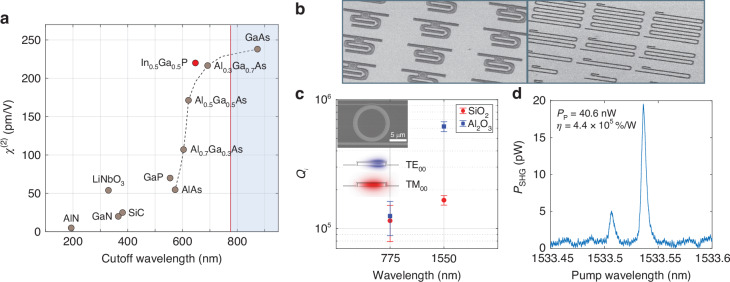
Loss optimization of InGaP photonic integrated circuits. **a** Second-order susceptibility and cutoff wavelength of several nonlinear optical thin-film materials. The red line indicates the 775 nm wavelength. Shaded region indicates optical absorption at wavelengths longer than 775 nm. AlN:^43^, LiNbO_3_:^44^, GaN:^45^, SiC:^46^, GaP:^47^, AlGaAs:^9^, GaAs:^47^, InGaP:^22^. **b** SEM images of InGaP photonic integrated circuits. **c** Intrinsic quality factor of the 1550 nm TE_00_ and 775 nm TM_00_ resonances of *R* = 5*μ*m InGaP microring resonators (inset) with SiO_2_ and Al_2_O_3_ claddings. Inset shows the electric field distribution of the two modes. **d** Second-harmonic generation in a *R* = 5*μ*m phase-matched InGaP microring resonator with Al_2_O_3_ cladding. On-chip pump power is 40.6 nW and the peak SHG efficiency is 440,000 %/W

Second-order (*χ*^(2)^) optical nonlinearity, as the dominant optical nonlinearity, enables a variety of nonlinear optical processes with high efficiencies and low noises, including the generation of entangled photons^4^ and squeezed light^5^, parametric optical amplification^6^, and coherent wavelength conversion^7^. Figure 1a displays the second-order susceptibility and cutoff wavelength of a selection of *χ*^(2)^ materials that are available in thin-film platforms. Among them, III-V semiconductors, including GaAs and Al_*x*_Ga_1−*x*_As, are notable for the very high second-order susceptibility, leading to a long history of study for nonlinear optics^8^. The versatile III-V photonics platform enables heteroeptaxial integration of pump lasers and photodetectors, which is unique compared to other platforms. However, one drawback of these III-V semiconductors is the optical losses at short wavelengths. For example, GaAs has a narrow bandgap corresponding to a cutoff wavelength of 872 nm. While Al_*x*_Ga_1−*x*_As exhibits a wider bandgap, its second-order susceptibility decreases drastically with the increasing aluminum composition^9^. Moreover, arsenic III-V materials suffer from strong optical absorption at wavelengths less than 800 nm, due to the antibonding As-As surface state that is below the bandgap^10–12^. These facts have limited the use of arsenic III-V materials for efficient second-order nonlinear optics in the important telecommunication C band (1530-1565 nm), where long-haul optical communications conducts, due to the absorption of the corresponding second harmonics.

Indium gallium phosphide (In_0.5_Ga_0.5_P, hereafter referred to as InGaP) is another III-V semiconductor material that is lattice-matched with GaAs and thus can be epitaxially grown on the GaAs substrate at the wafer scale. Because of its high electron mobility, direct bandgap, and thermal stability, InGaP has been used for making heterojunction bipolar transistors^13^, solar cells^14^, photodetectors^15^, and LEDs^16^. More recently, thin-film InGaP has been explored for third-order nonlinear optics using its substantial Kerr nonlinearity^17,18^, resulting in demonstrations of frequency combs^19^, optical parametric oscillators^20^, and entangled photon pairs via four-wave mixing^21^.

Besides its notable Kerr nonlinearity, InGaP is particularly appealing for second-order nonlinear optics because of the combination of a substantial second-order susceptibility (${\chi }_{xyz}^{(2)}\approx$ 220 pm/V^22^) and a sizable bandgap of 1.92 eV (cutoff wavelength 645 nm). For InGaP, the antibonding anion state lies well above the bandgap^11^, in contrast to Al_*x*_Ga_1−*x*_As, which avoids light absorption before the bandgap. InGaP also has a large refractive index ( > 3), which facilitates nanophotonic structures with strong light confinement. These properties suggest the potential of InGaP for realizing highly efficient second-order nonlinear optics, in particular, in the crucial telecommunication C band. Recently, several research groups have embarked on studying second-order nonlinear optics using thin-film InGaP^3,23,24^. Despite demonstrating a record nonlinearity-to-loss ratio in InGaP microring resonators^3^, most demonstrations thus far are still limited by considerable optical losses and imperfect phase-matching condition. Moreover, InGaP microcavities demonstrated in Ref. ^3^ are unsuitable for applications that demand broadband operation and high optical powers. As a result, realizing a broadband, low-loss, ultra-efficient second-order nonlinear photonics platform based on InGaP remains elusive.

Here, through the optimization of optical losses and phase-matching condition for InGaP nanophotonic waveguides across an octave wavelength span, we demonstrate a broadband, ultra-efficient InGaP second-order nonlinear photonics platform in the telecommunication band. The demonstrated second-harmonic generation with a normalized efficiency of 128,000%/W/cm^2^ in the telecommunication C band is nearly two orders of magnitude more efficient than the state of the art^25,26^. Using the InGaP nanophotonic waveguide, we demonstrate an ultra-bright time-energy entangled photon source with a pair generation rate of 97 GHz/mW and a bandwidth of 115 nm (14.4 THz) centered at the telecommunication C band. The broadband, ultra-efficient InGaP nanophotonics platform will enable a wide range of nonlinear optical processes and applications with unprecedented performances.

## Results

In this study, 110 nm thick InGaP is epitaxially grown on the GaAs substrate (0 degree off-cut toward [110]) using metal-organic chemical vapor deposition (T=545 C, V/III=280, with precursors including trimethylindium, trimethylgallium, and PH_3_). The root-mean-square (RMS) surface roughness of the InGaP thin film is measured to be about 0.3 nm, which is close to the native surface roughness of the GaAs substrate. To fabricate InGaP photonic integrated circuits, bonding of InGaP thin film to low-index substrates have been demonstrated before^24,27^. Here we adopted a transfer-free approach to fabricate InGaP photonic integrated circuits with low-index oxide top claddings (Methods)^3^. Figure 1b shows the scanning electron microscopy (SEM) images of fabricated InGaP photonic integrated circuits, including microring resonators and waveguides.

We studied the optical loss of InGaP nanophotonic devices in both 1550 nm and 775 nm wavelength bands with two different oxide claddings, SiO_2_ and Al_2_O_3_, deposited via atomic layer deposition. The optical loss is characterized using the intrinsic quality factor (${Q}_{i}\equiv \frac{\omega }{{\kappa }_{i}}$) of InGaP microring resonators. The microring resonator couples with both 1550 nm and 775 nm wavelength-band waveguides, which enable measurement of the transmission spectrum of the device^3^. *Q*_*i*_ is then inferred from the fitting of the resonance spectrum. Figure 1c shows the measured *Q*_*i*_ of the 1550 nm band fundamental transverse-electric (TE_00_) resonance and the 775 nm band fundamental transverse-magnetic (TM_00_) resonance of microring resonators with 5 *μ*m radius and 1 *μ*m width. The average value of *Q*_*i*,1550_ for microring resonators with Al_2_O_3_ cladding is about 6 × 10^5^, over three times higher than that with SiO_2_ cladding. *Q*_*i*,775_ is 1 − 2 × 10^5^ and shows slight improvement with Al_2_O_3_ cladding. The increase of the quality factor could be attributed to the surface passivation induced by Al_2_O_3_^28^. We also made microrings with different sizes and found *Q*_*i*,1550_ is peaked around 8 × 10^5^ for larger rings, which is limited by the absorption loss (see Section S2 of the Supplementary Information). Leveraging the optimized optical loss, we measured second-harmonic generation (SHG) in a 5-*μ*m-radius ring with phase-matched 1550 nm band TE_00_ and 775 nm band TM_00_ resonances and realized a resonant nonlinear conversion efficiency $\eta \equiv {P}_{{\rm{SHG}}}/{P}_{p}^{2}=440,000 \%$/W (Fig. 1d). This represents a 6-fold enhancement over the previously reported value using the same InGaP microring resonator but with SiO_2_ cladding^3^.

**Fig. 2:**
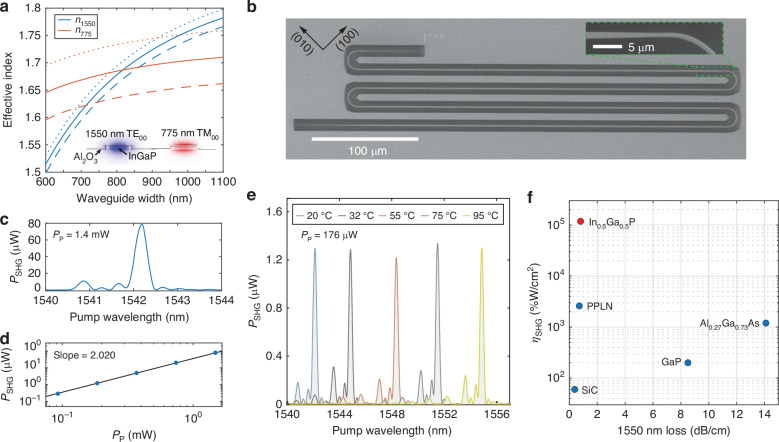
Second-harmonic generation. **a** Simulated effective mode index of 1550 nm TE_00_ and 775 nm TM_00_ modes of 108 nm (dashed), 110 nm (solid), and 112 nm (dotted) thick InGaP waveguides with 35 nm Al_2_O_3_ cladding. Inset is a cross section of the waveguide along with the electric field distribution of the two modes. **b** SEM images of a fabricated InGaP waveguide device. **c** Measured SHG spectrum for a 1.6 mm long waveguide device with a pump power of 1.56 mW. **d** Quadratic relation between the peak SHG power and the pump power. **e** Temperature tuning of the phase-matching condition. **f** Normalized SHG efficiency and 1550 nm wavelength band loss of the telecommunication C band waveguide SHG of several thin-film second-order nonlinear photonics platforms. InGaP: this work, PPLN:^25^, Al_0.27_Ga_0.73_As:^48^, GaP:^49^, SiC:^50^

In contrast to cavities, waveguides can be operated in the broadband regime and circumvent the light extraction issue associated with cavities. Similar to the microring resonator, the InGaP waveguide is designed for phase matching between the 1550 nm band TE_00_ mode *a* and the 775 nm band TM_00_ mode *b*, which satisfy 2*ω*_*a*_ = *ω*_*b*_ and 2*k*_*a*_ = *k*_*b*_. Lacking the birefringence in InGaP, phase matching is achieved by dispersion engineering of the InGaP nanophotonic waveguide. By designing the waveguide width, the effective index of the 1550 nm band TE_00_ mode and the 775 nm band TM_00_ mode can be equalized, as shown in Fig. 2a using finite element simulation. For the phase-matched waveguide with length *L*, the normalized SHG efficiency, ${\eta }_{{\rm{SHG}}}\equiv \frac{{P}_{{\rm{SHG}}}}{{P}_{p}^{2}{L}^{2}}$, can be calculated using^29^

1 $$\eta_{{\mathrm{SHG}}} = \frac{\omega_a^2}{2n_a^2n_b\epsilon_0 c^3} \left(\frac{{\int}\,{d}{\textbf{r}} \chi_{xyz}^{(2)} \sum\limits_{i\neq j\neq k}E_{bi}^*E_{aj}E_{ak}}{{\int} \,{d}{\textbf{r}}{|{\textbf{E}}_{a}|}^2\sqrt{{\int}\,{d}{\textbf{r}}{|{\textbf{E}}_b|}^2}} \right)^2$$

where *n*_*a*(*b*)_ is the effective mode index of the fundamental(second)-harmonic mode and the normalization integrals use electric field components perpendicular to the wavevector of the waveguide mode. The SHG efficiency is optimized when the waveguide is aligned along the (110) direction of InGaP, leading to simulated *η*_SHG_ = 130,000%/W/cm^2^ for the 1550 nm pump wavelength (see Section S3 of the Supplementary Information).

Figure 2b displays SEM images of a fabricated meander waveguide with a length of 1.6 mm. The waveguide connects to two adiabatically tapered couplers at the end to interface with tapered optical fibers for light transmission. The adiabatic coupler efficiency is approximately 80% and 30% for 1550 nm TE and 775 nm TM polarized light, respectively^3^. The waveguide width is tapered down before entering the 180° turn to avoid mode interference due to the bending of the multimode waveguide. The SHG nonlinear transfer function of the meander waveguide is given by ${\left(\frac{\sin N(x+\phi )}{N\sin (x+\phi )}\frac{\sin x}{x}\right)}^{2}$ (Methods), where $x=\frac{\Delta k{L}_{0}}{2}$, *Δk* = 2*k*_*ω*_ − *k*_2*ω*_, *L*_0_ is the length of the waveguide in one row, *N* is the number of rows, and 2*ϕ* is the total phase mismatch between the two modes in a 180° turn. For phase-matched fundamental- and second-harmonic modes in the straight waveguide, they become phase mismatched in the turn because of the change of the waveguide width and waveguide bending. A narrow waveguide section with a tunable length (Fig. 2b inset) is introduced to compensate the phase mismatch due to the bending waveguide such that the total phase mismatch 2*ϕ* through the 180° turn, including the tapering section, is multiple 2*π*. As a result, the transfer function can be recovered to the ideal sinc^2^ function.

A tunable continuous-wave telecom band laser is employed for the measurement of second-harmonic generation (laser output power 15 mW and linewidth <200 kHz (50 ms integration time)). The output light from the waveguide passes through a 1550 nm/775 nm wavelength division multiplexer (WDM) to filter the residual pump before the second-harmonic intensity is measured. Figure 2c shows the SHG intensity of a 1.6 mm long waveguide as the pump wavelength is swept, where a peak on-chip SH power *P*_SHG_ = 80*μ*W is observed for the pump wavelength 1542.1 nm and an on-chip pump power *P*_*p*_ = 1.56 mW. This corresponds to a normalized SHG efficiency of 128,000%/W/cm^2^. In comparison, normalized SHG efficiency of 2,500%/W/cm^2^ was achieved in lossy InGaP waveguides with phase-matched higher-order modes before^23^. A quadratic relationship between *P*_SHG_ and *P*_*p*_ is observed in the pump non-depleted region (Fig. 2d). Additionally, we explored the tunability of the waveguide’s phase-matching condition through temperature tuning of the device. In Fig. 2e, we present the measured SHG spectrum at several temperatures up to 95°C, constrained by the thermoelectric cooler element. A tuning range of 12.7 nm and a temperature-induced shift of 0.17 nm/°C in the phase-matching wavelength are measured. The redshift of phase-matching wavelength is consistent with the previously observed thermal-optical effect of the TE_00_ and TM_00_ modes of InGaP microrings^3^. The SHG efficiency is stable and continuous in the entire tuning range. The slight fluctuation seen in Fig. 2e is due to the re-alignment of fiber coupling for different temperature.

The SHG efficiency realized in the InGaP nonlinear nanophotonic waveguide represents a substantial advance, in particular, in the crucial telecommunication band. Figure 2f displays the normalized SHG efficiency and 1550 nm band loss of the best telecommunication C band waveguide SHG, to our knowledge, of several *χ*^(2)^ nonlinear photonics platforms (a more comprehensive list is provided in Section S7 of the Supplementary Information). The InGaP nanophotonic waveguide surpasses thin-film PPLN waveguides by nearly two orders of magnitude in terms of normalized nonlinear conversion efficiency^25,26^, while maintaining a low 1550 nm wavelength loss of 0.8 ± 0.4 dB/cm, which is consistent with the measured *Q*_*i*_ of microring resonators (equivalent to 0.4 dB/cm). The ratio of the normalized nonlinear efficiency between the InGaP and thin-film PPLN waveguides can be estimated using Eq. (1): ${(4\times 2\times \frac{\pi }{2})}^{2}\approx 160$, where 4 × is from ${\chi }_{xyz}^{(2)}$ of InGaP versus ${\chi }_{zzz}^{(2)}$ of LiNbO_3_, 2 × is from the swap of indices *x* and *y* in ${\chi }_{xyz}^{(2)}$ for the TE_00_ mode, and $1/\frac{2}{\pi }\times$ is due to the periodic poling of LiNbO_3_. On the other hand, the InGaP waveguide is slightly disadvantageous in terms of mode overlap because of the high-aspect-ratio cross section leading to less field confinement. The nonlinear efficiency of ${P}_{{\rm{SHG}}}/{P}_{p}^{2}=3280$%/W achieved in the 1.6 mm long InGaP waveguide is comparable to the centimeter-long PPLN waveguide made with the adapted poling technique recently^26^. For longer InGaP waveguides, we find the nonlinear efficiency deviates from the *L*^2^ scaling because of the thickness nonuniformity of the thin film (see Section S4 of the Supplementary Information). This can be mitigated using the adapted phase-matching technique^26^, by varying the waveguide width according to the pre-calibrated InGaP film thickness to keep the phase-matching condition along the entire waveguide.

Utilizing the InGaP nonlinear nanophotonic waveguide, we demonstrate an ultra-bright, broadband time-energy entangled photon source via spontaneous parametric down-conversion (SPDC). For a phase-matched nonlinear waveguide, the internal efficiency of the pair generation via SPDC can be related to the SHG efficiency by^30^

2 $$\begin{array}{rcl}{P}_{{\rm{SPDC}}}/{P}_{p}&\approx &\frac{\hslash {\omega }_{p}{L}^{3/2}}{3\sqrt{2\pi \left\vert {\rm{GVD}}\left(\frac{{\omega }_{p}}{2}\right)\right\vert }}{\eta }_{{\rm{SHG}}}\\ &=&\frac{\hslash {\omega }_{p}{L}^{2}\Delta {f}_{{\rm{FWHM}}}}{3\sqrt{2\pi }\alpha }{\eta }_{{\rm{SHG}}}\end{array}$$

where ${\rm{GVD}}\,\left(\frac{{\omega }_{p}}{2}\right)$ is the group velocity dispersion at $\frac{{\omega }_{p}}{2}$, $\alpha =\frac{1}{\pi }\sqrt{2{{\rm{sinc}}}^{-1}\frac{1}{\sqrt{2}}}$, and *Δf*_FWHM_ is the bandwidth of the SPDC photons given by

3 $$\Delta {f}_{{\rm{FWHM}}}=\frac{\alpha }{\sqrt{\left\vert {\rm{GVD}}\left(\frac{{\omega }_{p}}{2}\right)\right\vert L}}$$

**Fig. 3:**
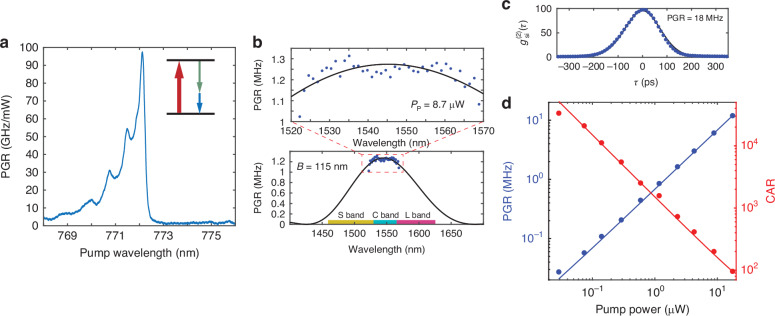
Spontaneous parametric down-conversion. **a** Dependence of the total SPDC pair generation rate (PGR) efficiency on the pump wavelength. **b** SPDC spectrum sampled by a DWDM with *P*_*p*_ = 8.7 μW. The solid line is a sinc^2^ function fitting, yielding a bandwidth of 115 nm (14.4 THz). **c** Measured ${g}_{{\rm{si}}}^{(2)}(\tau )$ for PGR = 18 MHz in one DWDM channel. Solid line is Gaussian fitting. **d** Pair generation rate in one DWDM channel and coincidence-to-accidental counts ratio (CAR) for various pump powers. Solid lines are linear fitting

We pumped the phase-matched waveguide with a tunable 780 nm band continuous-wave laser to generate telecommunication band SPDC photons. After filtering the residual pump, the SPDC photons were measured using either regular photodetectors or superconducting-nanowire single-photon detectors (SNSPDs). Figure 3a shows the total photon pair generation rate efficiency via a 1.6 mm long waveguide measured with *P*_*p*_ = 135*μ*W. At the phase-matching wavelength of 772.12 nm, a peak pair generation rate of 97 GHz/mW, corresponding to an internal efficiency of 2.5 × 10^−5^, is observed. To measure the bandwidth of the SPDC photons, we used a dense wavelength division-multiplexer (DWDM) with 40 channels and a 120 GHz channel bandwidth. The measured SPDC photon rate through each channel for *P*_*p*_ = 8.7*μ*W is displayed in Fig. 3b (the fiber coupling is not optimized for this measurement). The data is fitted using a sinc^2^ function and the SPDC photon bandwidth is inferred to be 14.4 THz (115 nm). The measured bandwidth agrees with the theoretical calculation using Eq. (3) (see Section S5 of the Supplementary Information). This leads to a per-bandwidth pair generation rate of 6.7 GHz/mW/THz (840 MHz/mW/nm). In Table 1, we compare the telecommunication C band SPDC photon pair generation in the InGaP waveguide and thin-film (TF) PPLN waveguides of several recent works. The InGaP waveguide SPDC source shows a rate efficiency at least an order of magnitude higher while retaining a large bandwidth. Since the per-bandwidth pair generation rate is ∝ *η*_SHG_*L*^2^, according to Eq. (2), such an enhancement is expected for the InGaP waveguide based on the normalized SHG efficiency. We also noticed a recent work of high-efficiency telecommunication L band SPDC photon generation in AlGaAs waveguides^12^. A more comprehensive summary of broadband photon pair sources can be found, for example, in Ref. ^31^.

**Table 1:** Telecommunication C band SPDC photon pair generation in TF PPLN and InGaP waveguides

Material	Rate (GHz/mW/THz)	Bandwidth (THz)	Waveguide length (mm)
TF PPLN^42^	0.12	–	10
TF PPLN^4^	0.46	–	5
TF PPLN^31^	0.13	100	5
InGaP (this work)	6.7	14.4	1.6

The second-order cross-correlation, ${g}_{{\rm{si}}}^{(2)}(\tau )$, between the signal and idler photons via two DWDM channels was measured using a pair of SNSPDs. Figure 3c displays the measured ${g}_{{\rm{si}}}^{(2)}(\tau )$ for pair generation rate of 18 MHz in one DWDM channel. In the low gain regime, the zero-delay cross-correlation between the signal and idler is shown to be^32^

4 $${g}_{{\rm{si}}}^{(2)}(0)=1+\frac{4B}{R}\frac{{\Gamma }_{s}{\Gamma }_{i}}{{({\Gamma }_{s}+{\Gamma }_{i})}^{2}}$$

where *R* and *B* are the total pair generation rate and bandwidth of the SPDC photons, respectively, and *Γ*_*s*,*i*_ is the filter bandwidth of the signal and idler photons. For *Γ*_*s*_ = *Γ*_*i*_, Eq. (4) indicates the inherent coincidence-to-accidental counts ratio (CAR) of the SPDC photon source is given by ${\rm{CAR}}={g}_{{\rm{si}}}^{(2)}(0)-1=B/R$, i.e., the inverse of the photon pair rate per bandwidth. According to Eq. (2) (*B* ≡ *Δf*_FWHM_ and *R* ≡ *P*_SPDC_/*ℏω*_*p*_), CAR ∝ 1/(*η*_SHG_*L*^2^*P*_*p*_), which means for more efficient waveguides, same CAR can be achieved with less pump power. Due to the detector jitter ( ~ 100 ps), which is much larger than the coherence time of the SPDC photons filtered by a DWDM channel (120 GHz), the inherent ${g}_{{\rm{si}}}^{(2)}(0)$ cannot be resolved and the measured CAR will be lower than the inherent value^32^. The measured CAR and pair generation rate in one DWDM channel for various pump power is shown in Fig. 3d (Methods). Nevertheless, CAR > 10^4^ is observed for relatively low photon pair generation rate.

**Fig. 4:**
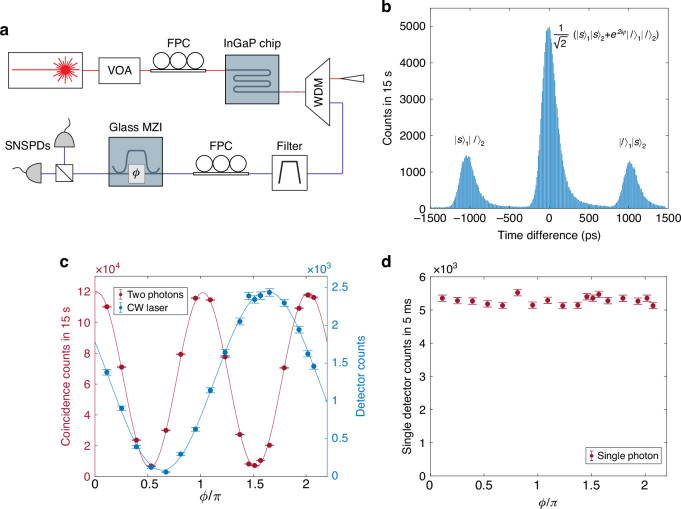
Time-energy entangled photon pair. **a** Schematic of the measurement of the time-energy entanglement of the SPDC photons. VOA: variable optical attenuator. FPC: fiber polarization controller. **b** Two-photon time difference histogram. The binwidth is 10 ps. The middle peak corresponds to the time-energy entangled state. **c** Coincidence counts in 800 ps binwidth integrated in 15 s of the SPDC photon pair for various interferometer phases (red) and the interference fringe of a CW laser (blue). Solid lines are sinusoidal function fitting. Error bars represent the shot noise. **d** Counts in 5 ms of SPDC photons in one SNSPD

To demonstrate the time-energy entanglement of the SPDC photons, we measured the two-photon interference using an unbalanced Mach-Zehnder interferometer (MZI)^33^, as illustrated in Fig. 4a (Methods). The unbalanced MZI is made from glass-based photonic integrated circuits with a path delay of *τ*_*d*_ = 1 ns. The SPDC photons used in this measurement have a bandwidth of about 20 nm as they are filtered by a CWDM, leading to a single-photon coherence time much shorter than *τ*_*d*_. The coherence time of the correlated signal-idler pair is determined by the continuous-wave pump laser, which is much longer than *τ*_*d*_. As a result, the signal-idler photon pair can interfere through the unbalanced MZI while neither can the signal or idler single photon. The signal-idler pair can travel through either the short (*s*) or long (*l*) path of the unbalanced MZI together, forming a time-energy entangled state $\left\vert \psi \right\rangle =\frac{1}{\sqrt{2}}({\left\vert s\right\rangle }_{1}{\left\vert s\right\rangle }_{2}+{e}^{2i\phi }{\left\vert l\right\rangle }_{1}{\left\vert l\right\rangle }_{2})$, where *ϕ* is the phase difference between the two paths for light with a frequency that is half of the SPDC pump frequency. This entangled state can be post-selected, distinguishing itself from the other two states out of the interferometer, ${\left\vert s\right\rangle }_{1}{\left\vert l\right\rangle }_{2}$ and ${\left\vert l\right\rangle }_{1}{\left\vert s\right\rangle }_{2}$, using time-resolved coincidence detection (Methods).

Figure 4b shows a measured time difference histogram of the SPDC photons. The coincidence counts around the zero delay, corresponding to the entangled state, depend on the interferometer phase as $\propto \frac{1}{2}(1+\cos 2\phi )$. Figure 4c shows the measured two-photon interference fringe by varying the temperature of the glass interferometer which changes *ϕ*. The two-photon fringe has a period of *π* while the fringe of a continuous-wave laser with a frequency about half of the pump frequency has a period of 2*π*. The counts of SPDC photons in one detector does not exhibit an interference fringe as expected (Fig. 4d). The measured two-photon interference visibility is 90.8%, which is limited by the imperfect glass photonic circuit MZI with the beam splitter ratio deviating from 50/50. The two-photon interference visibility is ≥98.6% after correction for the interferometer imperfection (see Section S6 of the Supplementary Information). The two-photon interference visibility exceeds the Clauser-Horne limit of $\frac{1}{\sqrt{2}}\approx 70.7 \%$, which proves the photon pair entanglement^34^.

## Discussion

In summary, we have demonstrated a broadband second-order nonlinear photonics platform based on thin-film InGaP. With the optimized optical loss and phase-matching condition, the InGaP nanophotonic waveguide enables second-order nonlinear optical processes, including SHG and SPDC, with normalized efficiencies one to two orders of magnitude higher than the state of the art in the telecommunication C band. The nonlinear efficiency of the InGaP waveguides can be further enhanced using the adapted fabrication technique^26^ to counter the thickness nonuniformity of thin films. The ultra-bright, broadband entangled photon pair source, covering the telecommunication C band, will be useful for high-rate wavelength-multiplexed entanglement distribution over long distances^35^ and ultrafast spectroscopy using entangled photons^36^. Beyond that, the demonstrated InGaP nonlinear photonics platform is expected to enable unprecedented performances in applications ranging from squeezed light generation^37,38^ and optical parametric amplification^39^ to few-photon quantum nonlinear optics^40^, among others. Based on III-V semiconductors, the InGaP platform also enables monolithic integration of pump lasers^41^ to realize electrically-injected nonlinear photonic sources.

## Method

### Device fabrication

The device fabrication follows Ref. ^3^. The device pattern is defined using electron beam lithography and transferred to InGaP layer via inductively coupled plasma reactive-ion etch (ICP-RIE) using a mixture of Cl_2_/CH_4_/Ar gas. Then a layer of aluminum oxide is deposited via atomic layer deposition. The InGaP device is released from the GaAs substrate using citric acid-based selective etching. See Section S1 of the Supplementary Information for more details.

### Measurement

For the measurement of SPDC photons, we used a 780 nm band continuous-wave tunable diode laser as the pump (output power 25 mW and linewidth < 200 kHz (50 ms integration time)). The light is polarization aligned by a fiber polarization controller, coupled into the device via a tapered fiber to generate the SPDC photons, and coupled back into the optical fiber using another tapered fiber. The residual pump is filtered by a 1550 nm/780 nm WDM. To measure the cross-correlation and CAR of the SPDC photons, the signal and idler photons are separated by a DWDM and detected using SNSPDs (Quantum Opus) and time-correlated single-photon counting module (Swabian). The coincidence and accidental counts are integrated in a time binwidth of 10 ps. To measure the time-energy entanglement of the SPDC photons, both signal and idler photons are filtered by the same CWDM channel and then pass through a glass waveguide unbalanced MZI (Teem Photonics). The two-photon coincidences are detected by two SNSPDs after the 50/50 beam splitter.

### Nonlinear transfer function

The transfer function of SHG is calculated as

5 $${\mathcal{F}}(L)={\left\vert \frac{1}{L}\mathop{\int}\nolimits_{\!\!0}^{L}{e}^{i\mathop{\int}\nolimits_{\!0}^{z}\Delta \phi dz^{\prime} }dz\right\vert }^{2}$$

where *Δϕ* is the phase mismatch between the fundamental- and second-harmonic modes. For a meander waveguide,

6 $$\begin{array}{rcl}{\mathcal{F}}(L)&=&{\left\vert \frac{1}{L}\sum\limits_{n}\mathop{\int}\nolimits_{n{L}_{0}}^{(n+1){L}_{0}}{e}^{i(\Delta kz+2n\phi )}dz\right\vert }^{2}\\ &=&{\left\vert \displaystyle\frac{{e}^{i\Delta k{L}_{0}}-1}{i\Delta kL}\frac{{e}^{iN(\Delta k{L}_{0}+2\phi )}-1}{{e}^{i(\Delta k{L}_{0}+2\phi )}-1}\right\vert }^{2}\\ &=&{\left(\displaystyle\frac{\sin (\Delta k{L}_{0}/2)}{\Delta kL/2}\frac{\sin \left(N(\Delta k{L}_{0}/2+\phi )\right)}{\sin (\Delta k{L}_{0}/2+\phi )}\right)}^{2}\end{array}$$

where *Δk* = 2*k*_*ω*_ − *k*_2*ω*_, *L*_0_ is the length of the waveguide in one row, *N* is the number of rows, and 2*ϕ* is the total phase mismatch between the two modes for a 180° turn.

## Supplementary information

Supplementary Information

## Data Availability

All data used in this study are available from the corresponding authors upon reasonable request.
