# Supplementary material for: InGaP χ(2) integrated photonics platform for broadband, ultra-efficient nonlinear conversion and entangled photon generation
Source: Light Sci Appl. 2024 Oct 15;13:290. doi: 10.1038/s41377-024-01653-5 (PMC11473533; doi:10.1038/s41377-024-01653-5)
Supplement: Supplementary file 1 — Supplementary Information [file 41377_2024_1653_MOESM1_ESM.pdf]

Supplementary Information for: "InGaP $\chi^{(2)}$ integrated photonics platform for broadband, ultra-efficient nonlinear conversion and entangled photon generation"

Joshua Akin,[1,2,†] Yunlei Zhao,[1,2,†] Yuvraj Misra,[1,2] A. K. M. Naziul Haque,[1,2] and Kejie Fang[1,2,*]

[1] *Holonyak Micro and Nanotechnology Laboratory and Department of Electrical and Computer Engineering, University of Illinois at Urbana-Champaign, Urbana, IL 61801 USA*

[2] *Illinois Quantum Information Science and Technology Center, University of Illinois at Urbana-Champaign, Urbana, IL 61801 USA*

S1. DEVICE FABRICATION

The waveguide devices are fabricated from 110 nm thick InGaP films grown on GaAs substrate (0 degree off-cut toward the [110]) by metal-organic chemical vapor deposition (T = 545 C, V/III = 280, precursors: trimethylindium, trimethylgallium and PH_3). The device pattern is defined using 150 keV electron beam lithography and 150 nm negative tone resist hydrogensilsesquioxane (HSQ). A 20 nm thick layer of silicon dioxide is deposited on InGaP via plasma-enhanced chemical vapor deposition (PECVD) to enhance the adhesion of HSQ. The pattern is transferred to InGaP via inductively coupled plasma reactive-ion etch (ICP-RIE) using $Cl_2/CH_4/Ar$ gas mixture with a selectivity of InGaP: HSQ: PECVD SiO_2 = 240: 90: 80. After a short buffered oxide etch to remove the residual oxide (both HSQ and PECVD oxide), a layer of 35 nm thick Al_2O_3 is deposited on the chip via atomic layer deposition. A second electron beam lithography and subsequent ICP-RIE using CHF_3 gas are applied to pattern etch-through holes in the Al_2O_3 layer for undercut of the InGaP device. Finally, the InGaP device is released from the GaAs substrate using citric acid-based selective etching. We blend 10 g citric acid monohydrate, 20.4 mL 30%wt hydrogen peroxide, and 47.1 mL DI water. Then we add 28 wt % ammonia solution drops until the pH is adjusted to 8.5. The pH during the etching is monitored by a pH meter.

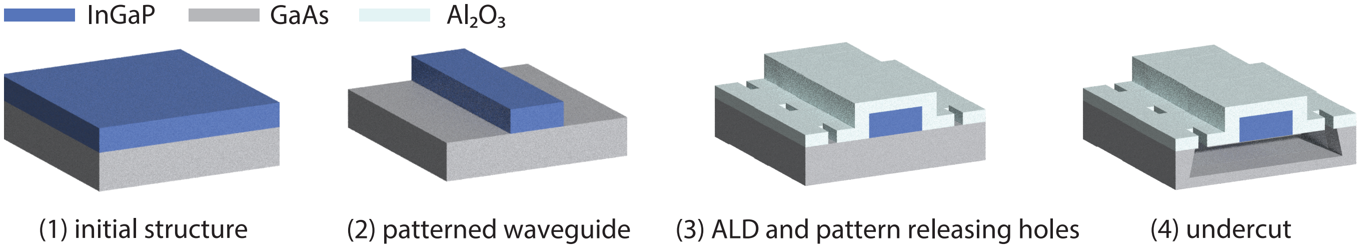

FIG. S1. Fabrication flow of InGaP waveguide devices.

S2. MICRORING QUALITY FACTOR

Microring resonators of different radius are made and the quality factor of the 1550 nm band TE_{00} resonances are measured. The result is displayed in Fig. S2. The intrinsic quality factor of the microring resonator is found to be bounded around 8×10^5, which is limited by the absorption loss, as the modeling below indicates.

The intrinsic quality factor of a microring resonator can be decomposed as $1/Q_i = 1/Q_r + 1/Q_{ss} + 1/Q_{sa} + 1/Q_{ba}$, where $1/Q_r$ is the radiation loss due to the curvature, $1/Q_{ss}$ is the surface roughness caused scattering loss, $1/Q_{sa}$ is the surface absorption loss, and $1/Q_{ba}$ is the bulk absorption loss. Below we follow Ref. [1] to calculate and model those loss components.

The splitting of the microring resonances due to surface roughness induced backscattering is given by

$$\frac{\Delta\lambda}{\lambda} = 2\sqrt{2}\pi^{3/4}\xi\frac{V_s}{V_r}, \tag{S1}$$

† These authors contributed equally to this work.
* kfang3@illinois.edu

where V_r is the physical volume of the microring, V_s is the effective volume of a typical scatterer, and ξ is the relative dielectric contrast constant defined as

$$\xi = \frac{\overline{n}^2(n_r^2 - n_0^2)}{n_r^2(\overline{n}^2 - n_0^2)}. \tag{S2}$$

n_r, n_0, and $\bar{n}$ are the indices of refraction for the InGaP microring, surrounding medium, and 2D effective slab, respectively.

The surface roughness induced scattering loss Q_{ss} is given by

$$Q_{ss} = \frac{3\lambda^3}{16\pi^{7/2} n_0 (n_r^2 - n_0^2)\xi} \frac{V_r}{V_s^2}. \tag{S3}$$

From Eqs. S1 and S3, we can calculate Q_{ss} using the measured resonance splitting $\Delta\lambda$. Q_{ss} is found to be on the order of 10^9 for all sizes of rings, which is much larger than the measured Q_i.

The surface absorption induced quality factor is found to be

$$Q_{sa} = \frac{\pi c V_r (\overline{n}^2 - n_0^2)}{2\lambda\gamma_{sa}\overline{n}^2 V_{sa}}, \tag{S4}$$

where $V_{sa} \propto 2\pi R$ denotes the surface volume inducing absorption. As a result, Q_{sa} is independent of R. We can also assume a constant bulk absorption loss. Finally, we fit the measured Q_i using $1/(1/Q_r + 1/Q_a)$ (since Q_{ss} are found to be much larger than Q_i), where Q_r is $\propto R^\alpha$ with α a fitting parameter and $1/Q_a \equiv 1/Q_{sa} + 1/Q_{ba}$ is a constant. The result is shown in Fig. S2.

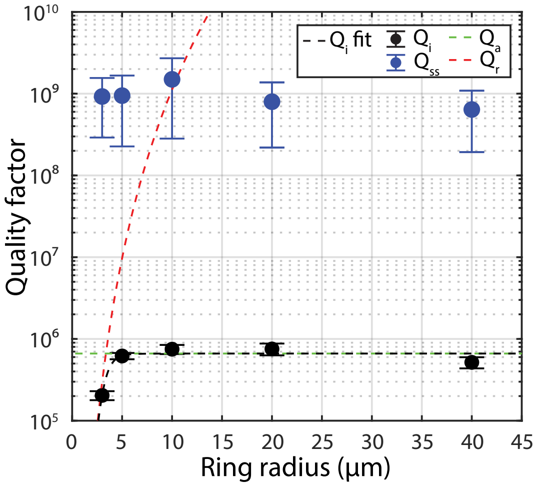

FIG. S2. Quality factor of the 1550 nm band TE_{00} resonances of microring resonators of different radius. Dashed lines are fitting. Q_{ss} are calculated from the splitting of resonances.

S3. SECOND HARMONIC GENERATION EFFICIENCY

For an InGaP waveguide with phase-matched fundamental-harmonic (FH) mode a and second-harmonic (SH) mode b, the SHG efficiency can be calculated using [2]:

$$\eta_{\text{SHG}} = \frac{\omega_a^2}{2n_a^2 n_b \epsilon_0 c^3} \left(\frac{\int d\mathbf{r}\chi_{xyz}^{(2)} \sum_{i\neq j\neq k} E_{bi}^* E_{aj} E_{ak}}{\int d\mathbf{r}|\mathbf{E}_a|^2 \sqrt{\int d\mathbf{r}|\mathbf{E}_b|^2}} \right)^2, \tag{S5}$$

with the normalization integrals only considering the transverse field components. Because the second-order susceptibility of InGaP only has the term $\chi^{(2)}_{xyz}$, the FH and SH modes are chosen to be TE_{00} and TM_{00}, respectively. Consider the orientation of the waveguide has an angle of θ with respect to the (100) direction of InGaP. We denote the electric field in the waveguide frame as $(E_{x'}, E_{y'}, E_{z'})$, where the $x' - z'$ plane is the waveguide cross-section plane and y' direction is along the waveguide. The electric field in the crystal frame is denoted as (E_x, E_y, E_z). As a result, $E_x = E_{x'}\cos\theta + E_{y'}\sin\theta$, $E_y = E_{x'}\sin\theta - E_{y'}\cos\theta$, and $E_z = E_{z'}$. Because the FH and SH modes are TE_{00} and TM_{00}, respectively, the dominant contribution to the numerator integral of Eq. S5 is given by

$$\begin{aligned}
&\int d\mathbf{r}\chi^{(2)}_{xyz}\sum_{i\neq j\neq k}E^*_{bi}E_{aj}E_{ak} \qquad \text{(S6)}\\
&= 2\int d\mathbf{r}\chi^{(2)}_{xyz}E^*_{bz}E_{ax}E_{ay}\\
&= 2\int d\mathbf{r}\chi^{(2)}_{xyz}E^*_{bz'}(E_{ax'}\cos\theta + E_{ay'}\sin\theta)(E_{ax'}\sin\theta - E_{ay'}\cos\theta)\\
&= \int d\mathbf{r}\chi^{(2)}_{xyz}E^*_{bz'}(E^2_{ax'} - E^2_{ay'})\sin 2\theta - 2\int d\mathbf{r}\chi^{(2)}_{xyz}E^*_{bz'}E_{ax'}E_{ay'}\cos 2\theta\\
&= \int d\mathbf{r}\chi^{(2)}_{xyz}E^*_{bz'}(E^2_{ax'} - E^2_{ay'})\sin 2\theta.
\end{aligned}$$

The second term in the second-last line is zero because $E_{ay'}$ is odd while $E_{ax'}$ and $E_{bz'}$ are even. Other terms contributing to the nonlinear interaction include

$$\begin{aligned}
&2\int d\mathbf{r}\chi^{(2)}_{xyz}E_{az}(E^*_{bx}E_{ay} + E^*_{by}E_{ax}) \qquad \text{(S7)}\\
&= 2\int d\mathbf{r}\chi^{(2)}_{xyz}E_{az'}(E^*_{bx'}\cos\theta + E^*_{by'}\sin\theta)(E_{ax'}\sin\theta - E_{ay'}\cos\theta)\\
&\quad +2\int d\mathbf{r}\chi^{(2)}_{xyz}E_{az'}(E^*_{bx'}\sin\theta - E^*_{by'}\cos\theta)(E_{ax'}\cos\theta + E_{ay'}\sin\theta)\\
&= 2\int d\mathbf{r}\chi^{(2)}_{xyz}E_{az'}(E^*_{bx'}E_{ax'}\sin 2\theta - E^*_{bx'}E_{ay'}\cos 2\theta - E^*_{by'}E_{ax'}\cos 2\theta - E^*_{by'}E_{ay'}\sin 2\theta)\\
&= 0,
\end{aligned}$$

which vanish because of the symmetry of the modes.

As a result, the mode overlap integral is maximized when $\theta = \frac{\pi}{4}$. The optimized SHG efficiency thus is given by

$$\eta_{\mathrm{SHG}} = \frac{\omega_a^2}{2n_a^2 n_b \epsilon_0 c^3}\left(\frac{\int d\mathbf{r}\chi^{(2)}_{xyz}E^*_{bz'}(E^2_{ax'} - E^2_{ay'})}{\int d\mathbf{r}|\mathbf{E}_a|^2\sqrt{\int d\mathbf{r}|\mathbf{E}_b|^2}}\right)^2. \qquad \text{(S8)}$$

The E field of the FH and SH modes are obtained by finite element simulation and the optimized SHG efficiency for 110 nm thick InGaP phase-matched waveguide and 1550 nm FH wavelength is found to be $\eta_{\mathrm{SHG}} = 130,000\%/\mathrm{W/cm}^2$, which agrees with the measured value. Fig. S3 shows the simulated field distribution of the FH and SH modes and the calculated mode overlap integrand $\chi^{(2)}_{xyz}E^*_{bz'}(E^2_{ax'} - E^2_{ay'})$.

S4. NONLINEAR EFFICIENCY OF INGAP WAVEGUIDES

The nonlinear efficiency of long InGaP waveguides is limited by the thickness nonuniformity of the thin film. Fig. S4a-c display the SHG spectrum of three waveguides with length 0.8, 1.6, and 3.2 mm, respectively, measured with $P_p = 1.56$ mW. It is observed that the spectrum of the 3.2 mm long waveguide distorts more due to the thickness nonuniformity as well as the phase mismatch at the 180° waveguide turn. Fig. S4d shows the peak nonlinear efficiency of the three waveguides. The nonlinear efficiency of the 3.2 mm long waveguide deviates from the L^2 scaling.

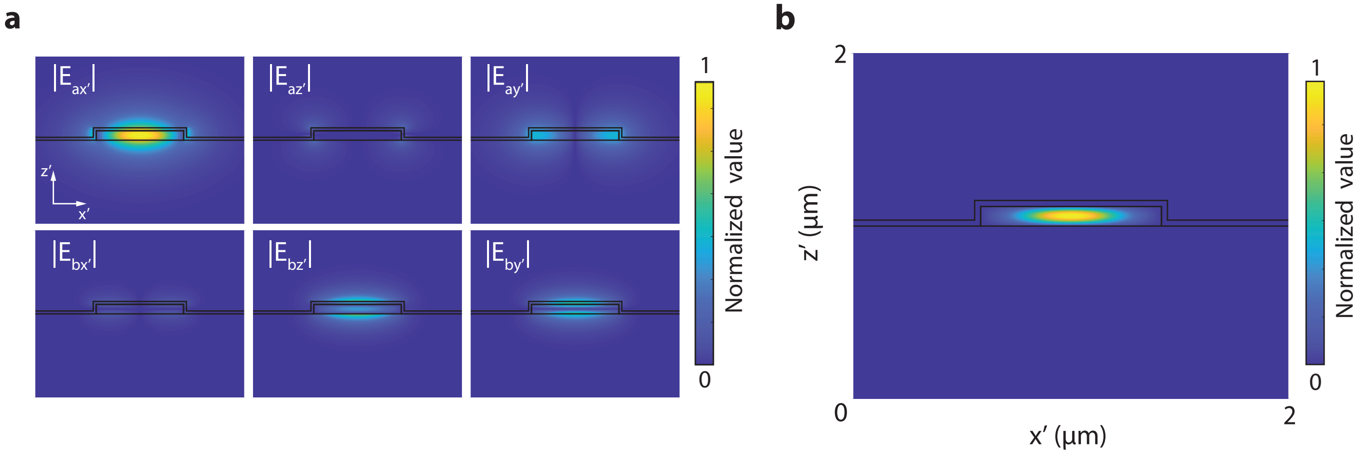

FIG. S3. **a**. Field distribution of the 1550 nm TE_{00} and 775 nm TM_{00} waveguide modes. **b**. Mode overlap integrand $\chi^{(2)}_{xyz}E^*_{bz'}(E^2_{ax'} - E^2_{ay'})$.

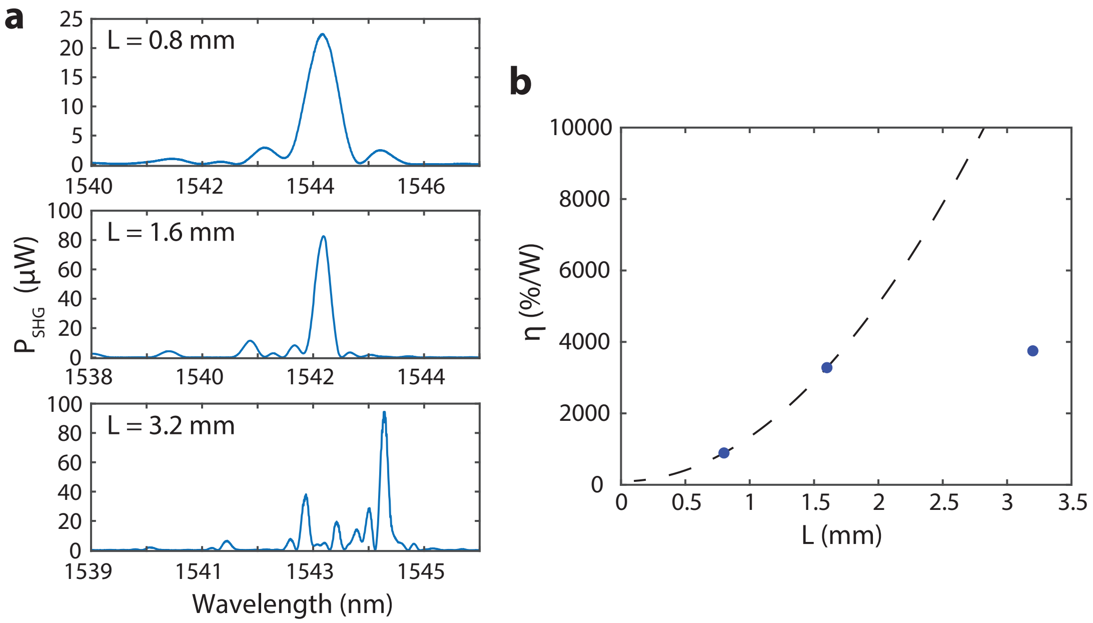

FIG. S4. **a-c**. Measured SHG spectrum of three waveguides with length 0.8, 1.6, and 3.2 mm, respectively. **d**. Peak nonlinear efficiency v. waveguide length. Dashed line indicates the L^2 scaling.

S5. BANDWIDTH OF WAVEGUIDE SPDC PHOTONS

The SPDC signal power in a bandwidth of $d\omega_s$ is given by [3]

$$dP_s = \frac{\hbar d_{\text{eff}}^2 P_p \omega_s^2 \omega_i L^2}{\pi \epsilon_0 c^3 n_s n_i n_p A_{\text{eff}}} \text{sinc}^2\left(\frac{\Delta k L}{2}\right) d\omega_s, \tag{S9}$$

where the d_{eff} is the nonlinear coefficient, L is the waveguide length, P_p is the pump power, A_{eff} is the effective interaction area, and Δk is the phase mismatch between the pump, signal and idler defined as

$$\Delta k = \frac{(n_p\omega_p - n_s\omega_s - n_i\omega_i)}{c}. \tag{S10}$$

Eq. S9 shows the ideal sinc^2 function of the SPDC spectrum and the bandwidth of the SPDC photons can be calculated from Eq. S10.

The InGaP waveguide is designed to be perfectly phase matched for the degenerate signal and idler. Thus the nondegenerate signal and idler frequencies are expressed most conveniently as

$$\omega_{s,i} = \frac{\omega_p}{2} \pm \Delta\omega. \tag{S11}$$

Using Eq. S10, the phase mismatch can be expressed in terms of the group velocity dispersion:

$$\Delta k = \mathrm{GVD}\left(\frac{\omega_p}{2}\right)\Delta\omega^2, \tag{S12}$$

where the group velocity dispersion is defined as

$$\mathrm{GVD}(\omega_0) \equiv \frac{2}{c}\left(\frac{\partial n}{\partial \omega}\right)_{\omega=\omega_0} + \frac{\omega_0}{c}\left(\frac{\partial^2 n}{\partial \omega^2}\right)_{\omega=\omega_0}. \tag{S13}$$

$n(\omega)$ is the effective mode index of the waveguide mode corresponding to the signal and idler. Using Eqs. S9 and S12, the full-width-half-maximum (FWHM) bandwidth of the SPDC spectrum is then given by

$$\Delta f_{\mathrm{FWHM}} = \frac{\alpha}{\sqrt{|\mathrm{GVD}\left(\frac{\omega_p}{2}\right)|L}}, \tag{S14}$$

where $\alpha = \frac{1}{\pi}\sqrt{2\mathrm{sinc}^{-1}\frac{1}{\sqrt{2}}}$.

For the waveguide used for the SPDC process, $L = 1.6$ mm and the pump frequency is $\omega_p/2\pi = 388.55$ THz. We simulated the dispersion of the effective mode index of the TE_{00} waveguide mode around $\omega_p/2$ and found $|\mathrm{GVD}\left(\frac{\omega_p}{2}\right)| = 0.373\ (\mathrm{ps})^2\mathrm{m}^{-1}$. This yields a SPDC bandwidth of 12.1 THz, which is close to the measured value.

S6. TWO-PHOTON INTERFERENCE VISIBILITY

Consider an unbalanced Mach-Zehnder interferometer (MZI) consisting of two beam splitters, with transmission and reflection coefficients $T_{1(2)}$ and $R_{1(2)}$, which satisfy $T_k^2 + R_k^2 = 1$. For a single-photon input state, the output state of the MZI is given by

$$|1\rangle \rightarrow T_1T_2|s\rangle + R_1R_2e^{i\phi}|l\rangle, \tag{S15}$$

where $|s\rangle$ and $|l\rangle$ represent the state in the short and long arm of the unbalanced MZI, respectively, and ϕ is the phase difference of the two arms. The visibility of the single-photon interference fringe thus is

$$V_1 = \frac{2T_1T_2R_1R_2}{T_1^2T_2^2 + R_1^2R_2^2}. \tag{S16}$$

The single-photon interference visibility can be characterized using a CW laser.

For a correlated two-photon input state $|1\rangle_1|1\rangle_2$ generated via SPDC, without parasitic noises, the output state thus is given by

$$|1\rangle_1|1\rangle_2 \rightarrow T_1^2T_2^2|s\rangle_1|s\rangle_2 + T_1T_2R_1R_2e^{i\phi}|s\rangle_1|l\rangle_2 + T_1T_2R_1R_2e^{i\phi}|l\rangle_1|s\rangle_2 + R_1^2R_2^2e^{2i\phi}|l\rangle_1|l\rangle_2, \tag{S17}$$

where ϕ is the interferometer phase difference corresponding to half of the SPDC pump frequency. In this output state, only the first and last terms contribute to the two-photon interference, since they cannot be distinguished in the two-photon coincidence measurement. The visibility of the two-photon interference is given by

$$V_2 = \frac{2T_1^2T_2^2R_1^2R_2^2}{T_1^4T_2^4 + R_1^4R_2^4}. \tag{S18}$$

Using Eqs. S16 and S18, the two interference visibilities are related by

$$V_2 = \frac{2}{\frac{4}{V_1^2} - 2}. \tag{S19}$$

We model the measured two-photon interference visibility $V_{2,m}$ deviating from the ideal case (Eq. S19) using some uncorrelated counts x. We stress this is an effective model and it could represent other physcal imperfection in the setup. The minimum and maximum two-photon coincidence counts are given by $C_{\min} = (T_1^2 T_2^2 - R_1^2 R_2^2)^2 + x$ and $C_{\max} = (T_1^2 T_2^2 + R_1^2 R_2^2)^2 + x$. The measured two-photon interference visibility thus is

$$V_{2,m} = \frac{2T_1^2 T_2^2 R_1^2 R_2^2}{T_1^4 T_2^4 + R_1^4 R_2^4 + x}. \tag{S20}$$

Using Eqs. S16 and S20, we can find the bound of x:

$$\begin{aligned} x &= \frac{2}{V_{2,m}} T_1^2 T_2^2 R_1^2 R_2^2 - T_1^4 T_2^4 - R_1^4 R_2^4 \\ &= T_1^2 T_2^2 R_1^2 R_2^2 \left(\frac{2}{V_{2,m}} - \frac{4}{V_1^2} + 2 \right) \\ &= \mu^2 \left(\frac{(1 - R_1^2) R_1^2}{(\mu^2 - 1) R_1^2 + 1} \right)^2 \left(\frac{2}{V_{2,m}} - \frac{4}{V_1^2} + 2 \right) \\ &\le \frac{\mu^2}{(\mu + 1)^4} \left(\frac{2}{V_{2,m}} - \frac{4}{V_1^2} + 2 \right) \\ &= x_{\max}, \end{aligned} \tag{S21}$$

where $\mu = (1 + \sqrt{1 - V_1^2})/V_1$. In our experiment, the measured CW interference visibility and two-photon interference visibility are $V_1 = 97.9\%$ and $V_{2,m} = 90.8\%$, respectively, from which we find $x_{\max} = 0.00179$.

We can then estimate the inherent two-photon interference visibility, $V_{2,i}$, if we were to use a perfect unbalanced MZI with $T_k^2 = R_k^2 = \frac{1}{2}$,

$$V_{2,i} = \frac{\frac{1}{8}}{\frac{1}{8} + x} \ge \frac{\frac{1}{8}}{\frac{1}{8} + x_{\max}} = 98.6\%. \tag{S22}$$

If we were to use a practically better interferometer with $V_1 = 99.8\%$ (achieved with $T_k^2 = 0.484$ and $R_k^2 = 0.516$), then using Eq. S20 and the estimated bound $x_{\max}$, the measured two-photon interference visibility will be $V_{2,m} \ge 97.8\%$.

S7. WAVEGUIDE SHG IN THIN-FILM $\chi^{(2)}$ PHOTONICS PLATFORMS

In Table S1, we summarize results, to our knowledge, of thin-film waveguide SHG in the telecommunication and near-infrared wavelength bands from recent works. The top half of the table before the break is in the telecommunication C band.

TABLE S1. **Thin-film waveguide SHG performances**

Material	$\chi^{(2)}$ (pm V^{-1})	poling	pump wavelength (nm)	η_{SHG} ($W^{-1}cm^{-2}$)	η (W^{-1})	FH loss (dB cm^{-1})
InGaP (this work)	$\chi^{(2)}_{xyz} = 220$	no	1550	128000%	3280%	0.8
InGaP [4]	$\chi^{(2)}_{xyz} = 220$	no	1536	2500%	12%	12
PGLN [5]	$\chi^{(2)}_{zzz} = 54$	no	1550	60%	31%	3
LN [2]	$\chi^{(2)}_{zzx} = 8.6$	no	1540	22%	5%	0.5
PPLN [6]	$\chi^{(2)}_{zzz} = 54$	yes	1550	2600%	42%	~ 1
PPLN [7]	$\chi^{(2)}_{zzz} = 54$	yes	1544	1000%	660%	0.7
PPLN [8]	$\chi^{(2)}_{zzz} = 54$	yes	1530	2000%	9000%	0.8
$Al_{0.27}Ga_{0.73}As$ [9]	$\chi^{(2)}_{xyz} = 210$	no	1560	1202%	87%	14
$Al_{0.19}Ga_{0.81}As$ [10]	$\chi^{(2)}_{xyz} = 220$	no	1590	1600%	16%	15
$Al_{0.2}Ga_{0.8}As$ [11]	$\chi^{(2)}_{xyz} = 210$	no	1580	23000%	477%	≤ 2
GaP [12]	$\chi^{(2)}_{yxx} = 70$	yes	1595	200%	14%	8
SiC [13]	$\chi^{(2)}_{zzz} = 25$	no	1584	60%	5%	0.4 [14]
GaAs [15]	$\chi^{(2)}_{xyz} = 240$	no	2025	13000%	250%	2
GaAs [16]	$\chi^{(2)}_{xyz} = 240$	no	1968	47600%	4000%	1.5

Supplementary information accompanies the manuscript on the Light: Science & Applications website (http://www.nature.com/lsa).

[1] Borselli, M., Johnson, T. J. & Painter, O. Beyond the Rayleigh scattering limit in high-Q silicon microdisks: theory and experiment. *Optics Express* **13**, 1515–1530 (2005).

[2] Luo, R., He, Y., Liang, H., Li, M. & Lin, Q. Highly tunable efficient second-harmonic generation in a lithium niobate nanophotonic waveguide. *Optica* **5**, 1006–1011 (2018).

[3] Kumar, R. & Ghosh, J. Parametric down-conversion in ppLN ridge waveguide: a quantum analysis for efficient twin photons generation at 1550 nm. *Journal of Optics* **20**, 075202 (2018).

[4] Poulvellarie, N. *et al.* Efficient type II second harmonic generation in an indium gallium phosphide on insulator wire waveguide aligned with a crystallographic axis. *Optics Letters* **46**, 1490–1493 (2021).

[5] Wang, C. *et al.* Second harmonic generation in nano-structured thin-film lithium niobate waveguides. *Optics Express* **25**, 6963–6973 (2017).

[6] Wang, C. *et al.* Ultrahigh-efficiency wavelength conversion in nanophotonic periodically poled lithium niobate waveguides. *Optica* **5**, 1438–1441 (2018).

[7] Stokowski, H. S. *et al.* Integrated quantum optical phase sensor in thin film lithium niobate. *Nature Communications* **14**, 3355 (2023).

[8] Chen, P.-K. *et al.* Adapted poling to break the nonlinear efficiency limit in nanophotonic lithium niobate waveguides. *Nature Nanotechnology* **19**, 44–50 (2024).

[9] May, S., Kues, M., Clerici, M. & Sorel, M. Second-harmonic generation in AlGaAs-on-insulator waveguides. *Optics Letters* **44**, 1339–1342 (2019).

[10] Roland, I. *et al.* Second-harmonic generation in suspended AlGaAs waveguides: A comparative study. *Micromachines* **11**, 229 (2020).

[11] Placke, M. *et al.* Telecom-Band Spontaneous Parametric Down-Conversion in AlGaAs-on-Insulator Waveguides. *Laser & Photonics Reviews* 2301293 (2024).

[12] Pantzas, K. *et al.* Continuous-wave second-harmonic generation in orientation-patterned gallium phosphide waveguides at telecom wavelengths. *ACS Photonics* **9**, 2032–2039 (2022).

[13] Zheng, Y. *et al.* Efficient second-harmonic generation in silicon carbide nanowaveguides. In *2022 Conference on Lasers and Electro-Optics (CLEO)*, 1–2 (IEEE, 2022).

[14] Ou, H. *et al.* Novel Photonic Applications of Silicon Carbide. *Materials* **16**, 1014 (2023).

[15] Chang, L. *et al.* Heterogeneously integrated GaAs waveguides on insulator for efficient frequency conversion. *Laser & Photonics Reviews* **12**, 1800149 (2018).

[16] Stanton, E. J. *et al.* Efficient second harmonic generation in nanophotonic GaAs-on-insulator waveguides. *Optics Express* **28**, 9521–9532 (2020).
